# Gene Therapy Developments for Pompe Disease

**DOI:** 10.3390/biomedicines10020302

**Published:** 2022-01-28

**Authors:** Zeenath Unnisa, John K. Yoon, Jeffrey W. Schindler, Chris Mason, Niek P. van Til

**Affiliations:** 1 AVROBIO, Inc., Cambridge, MA 02139, USA; zeenath.unnisa@avrobio.com (Z.U.); john.yoon@avrobio.com (J.K.Y.); rena.schindler@avrobio.com (J.W.S.); chris.mason@avrobio.com (C.M.); 2 Advanced Centre for Biochemical Engineering, University College London, London WC1E 6BT, UK; 3 Child Neurology, Emma Children’s Hospital, Amsterdam University Medical Centers, Vrije Universiteit and Amsterdam Neuroscience, 1081 HV Amsterdam, The Netherlands

**Keywords:** Pompe disease, enzyme replacement therapy, adeno-associated viral vector, hematopoietic stem cell, lentiviral vectors

## Abstract

Pompe disease is an inherited neuromuscular disorder caused by deficiency of the lysosomal enzyme acid alpha-glucosidase (GAA). The most severe form is infantile-onset Pompe disease, presenting shortly after birth with symptoms of cardiomyopathy, respiratory failure and skeletal muscle weakness. Late-onset Pompe disease is characterized by a slower disease progression, primarily affecting skeletal muscles. Despite recent advancements in enzyme replacement therapy management several limitations remain using this therapeutic approach, including risks of immunogenicity complications, inability to penetrate CNS tissue, and the need for life-long therapy. The next wave of promising single therapy interventions involves gene therapies, which are entering into a clinical translational stage. Both adeno-associated virus (AAV) vectors and lentiviral vector (LV)-mediated hematopoietic stem and progenitor (HSPC) gene therapy have the potential to provide effective therapy for this multisystemic disorder. Optimization of viral vector designs, providing tissue-specific expression and GAA protein modifications to enhance secretion and uptake has resulted in improved preclinical efficacy and safety data. In this review, we highlight gene therapy developments, in particular, AAV and LV HSPC-mediated gene therapy technologies, to potentially address all components of the neuromuscular associated Pompe disease pathology.

## 1. Introduction

Pompe disease is a rare autosomal recessive metabolic disorder caused by mutations in the enzyme coding for acid alpha-glucosidase (GAA) protein [1]. GAA enzyme is active in the acidic milieu of lysosomes and breaks down glycogen to glucose in cellular lysosomes. A mutation in this enzyme causes accumulation of glycogen resulting in a broad spectrum of clinical manifestations determined by the degree of protein dysfunction observed in skeletal, respiratory and cardiac muscles [2]. Among the pathophysiological changes, neuroinflammation is the hallmark of central nervous system (CNS) involvement leading to neurodegeneration and cognitive impairment [3]. Apart from lysosomal dysfunction, the accumulation of glycogen also results in autophagic build-up in muscle fibers that may later result in resistance to treatment approaches [4]. Pompe disease is broadly classified into two main categories consisting of infantile-onset Pompe disease (IOPD) and late-onset Pompe disease (LOPD), which is largely dependent on residual GAA enzyme activity. IOPD is diagnosed at birth and results in death by cardiorespiratory failure within the first year of life if untreated [2]. LOPD has a slower progression of muscle deterioration leading to motor impairment and ultimately respiratory failure [5,6]. Intravenous (IV) enzyme replacement therapy (ERT) is the standard of care aiming to restore the intracellular GAA enzyme activity and address the cellular pathology [7]. However, ERT has a risk of immunological responses, has poor uptake in target tissues, and does not reach the CNS because of the blood–brain barrier (BBB). Novel developments- in this field predominately include adeno-associated viral (AAV) gene therapy and lentiviral (LV) gene therapy in the context of hematopoietic stem and progenitor cell (HSPC) transplantation (HSCT). Other gene-modifying applications in the preclinical stage are also briefly discussed in this review, along with the challenges associated with each method. An overview of current preclinical, clinical stage, and marketed therapies are presented in Table 1.

## 2. Current Standard of Care for Pompe Disease

### Enzyme Replacement Therapy

The current standard of care for Pompe disease is ERT, which requires lifelong IV administration of recombinant human GAA (rhGAA). Presently, alglucosidase alfa (Myozyme^®^ or Lumizyme^®^) is the only rhGAA approved treatment for both IOPD and LOPD. The dosing regimen of 20 mg/kg biweekly is relatively high compared to other lysosomal storage disease (LSD) ERTs (Table 2), and although this dosing regimen achieves effective glycogen clearance in the heart and resolution of cardiomyopathy [8], it is less effective at glycogen resolution in skeletal muscle and does not resolve respiratory or neurological decline [9].

One of the main challenges of providing long-term symptom-free survival using ERT alone is the limited efficiency in delivering rhGAA to affected tissues [11], which is due to a combination of factors including low concentrations of rhGAA in the interstitial space, a small percentage of rhGAA containing the necessary mannose-6-phosphate (M6P) modifications needed for binding the cation-independent mannose 6-phosphate receptor (CI-M6PR), a low abundance of CI-M6PR on skeletal muscle cells, abnormal M6P trafficking in lysosomal storage diseases [12] and a preferential uptake of rhGAA in the liver leading to non-productive clearance of the enzyme.

GAA protein maturation proceeds through a precursor form with a molecular weight of 110kDa [13], which after intracellular proteolytic maturation and glycosylation in the Golgi network leads to the active mature form of 70–76 kDa found in the lysosome [14]. In healthy individuals, lysosomal GAA breaks down glycogen into glucose. However, efficient lysosomal targeting of rhGAA requires binding of M6P moieties to CI-M6PR expressed on the cell surface [15,16]. CI-M6PR, also known as insulin-like growth factor receptor (IGF2R), is a polyfunctional transmembrane protein with high affinity for M6P [15].

Because of the inherent limitations of rhGAA delivery to affected tissues, protein modifications for improving cellular uptake and bioavailability of rhGAA have been developed. Two approaches to increase rhGAA binding to CI-M6PR include the addition of phosphorylated oligosaccharide moieties [17,18], which is the strategy employed in the second-generation ERT avalglucosidase alfa (Nexviazyme^®^) recently approved for LOPD with ongoing clinical trials for IOPD [NCT03019406]), and the addition of a glycosylation-independent lysosomal targeting (GILT) peptide tag that consists of a portion of insulin-like growth factor 2 (IGF2) to increase the binding affinity of rhGAA to the IGF2 binding site of IGF2R/CI-M6PR (BioMarin Pharmaceutical). An investigational study was performed on safety, tolerability, pharmacokinetics, and pharmacodynamics of GILT-tagged rhGAA (NCT01230801) and a phase 3 study in LOPD patients was initiated (NCT01924845). Although the study demonstrated improvements in respiratory muscle strength, lung function, and walking endurance in subjects with LOPD, the sponsor discontinued the program for reasons unrelated to concerns for patients’ safety or drug effectiveness.

In preclinical studies, Pompe mice treated with neo-rhGAA, engineered to include an increased number of M6P residues, showed a significantly higher reduction of glycogen in skeletal muscle compared to mice treated with rhGAA. These mice also showed a comparable reduction of glycogen in the heart and diaphragm at a 4-fold lower dose [17]. Furthermore, mice treated with neo-rhGAA showed improvement in motor function in both rotarod and wire hang tests, although this improvement was not seen in older Pompe mice. In a randomized double-blind phase 3 trial (NCT02782741) comparing the glycoengineered avalglucosidase with the existing standard of care, alglucosidase, patients treated with avalglucosidase showed a clinically relevant improvement in respiratory function compared to patients treated with alglucosidase, as well as additional improvement in functional endurance and muscle strength [19].

Yet another innovative approach to increase the bioavailability and improve the pharmacokinetics and pharmacodynamics of rhGAA is the use of small molecule chaperones to stabilize and enhance the GAA enzymatic activity. Chaperones accomplish this by aiding in the proper folding of GAA protein which allows it to retain its catalytic activity and prevent premature degradation in the endoplasmic reticulum. These chaperones can be given in tandem with conventional ERT [20], or in the context of a novel rhGAA with higher M6P levels [21]. In addition to the correction of the glycogen buildup in skeletal muscle in a preclinical Pompe mouse model, the combination of the novel rhGAA (ATB200) co-administered with the small molecule chaperone AT2221 (miglustat, N-butyl-deoxynojirimycin [NB-DNJ]) was able to decrease autophagic accumulation in skeletal muscle and improve both wire hang and grip test function in a preclinical mouse model, however, clinical trial results failed to show superiority over the existing standard of care and suggest further long-term studies are needed (NCT03729362) [22].

Other novel rhGAA delivery approaches have been evaluated for safety and efficacy, including VAL-1221 (Valerion Therapeutics) which is a fusion protein of monoclonal lupus anti DNA-antibody 3E10 with rhGAA, allowing the modified rhGAA to enter the cell via the equilibrative nucleoside transporter 2 (ENT2), independent of the CI-M6PR. ENT2 is expressed at high levels in skeletal muscles and heart [23]. However, this trial was terminated by the sponsor in 2020 due to a lack of funding.

rhGAA does not efficiently cross the BBB. In order to achieve this, strategies exploiting receptor-mediated endocytosis and transcytosis pathways via receptors endogenously expressed at the brain capillary endothelium have been explored [24]. Targeting the transferrin receptor (TfR) by fusing rhGAA to an anti-human transferrin receptor antibody enables transcytosis across the BBB and delivery into the CNS compartment [25]. In addition to the limited ability of current ERT regimens to penetrate into the CNS, another limitation of ERT is the risk of immunological response. A higher dosage of ERT was beneficial for glycogen clearance from skeletal muscle but resulted in the formation of high-titer anti-rhGAA IgG antibodies in cross-reacting immunological material (CRIM) negative Pompe patients [26]. This poor response in CRIM negative Pompe patients is likely due to the complete absence of GAA protein, which would therefore not develop immune tolerance. Mice lacking *Gaa* expression also develop severe immune responses to rhGAA infusions [27].

**Figure 1:**
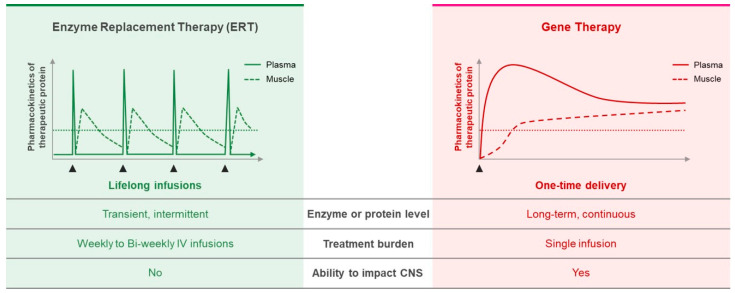
Anticipated pharmacokinetics of enzyme replacement therapy (ERT) using recombinant human GAA (rhGAA) protein and single intervention gene therapy. Left: ERT requires intermittent bolus infusions of doses of rhGAA protein to reach above the critical threshold. It has been reported that above 30% GAA enzyme activity present in unaffected individuals is the critical threshold [1]. Peak plasma rhGAA protein levels are present directly after infusion, subsequently taken up by muscles, and degraded over time. Right: Gene therapy applied as a single intervention therapy for curative potential. After transduction of cells of interest, continuous production of therapeutic transgene product provides sustained levels in transduced cells and/or secreted levels in plasma for cross-correction in key tissues. Application of gene therapy may impact the bioavailability of therapeutic enzymes to enhance uptake and correction in key tissues compared to ERT as shown by Costa-Verdera and colleagues [28]. Horizontal dotted line represents the critical threshold to prevent Pompe disease phenotype.

The advancement of second-generation ERT is likely to improve the efficacy in Pompe patients; however, the inherent challenges and limitations such as repeated life-long infusions, immunological reactions and the inability of rhGAA to cross the BBB in Pompe patients with CNS pathologies remain. Nevertheless, some GAA protein modifications that improve secretion and cellular uptake may be incorporated into single treatment modalities such as gene therapy, which would provide constant expression levels above the critical threshold needed to prevent or correct muscle and CNS pathology directly or through cross-correction (Figure 1).

## 3. Gene Therapy for Pompe Disease

**Figure 2:**
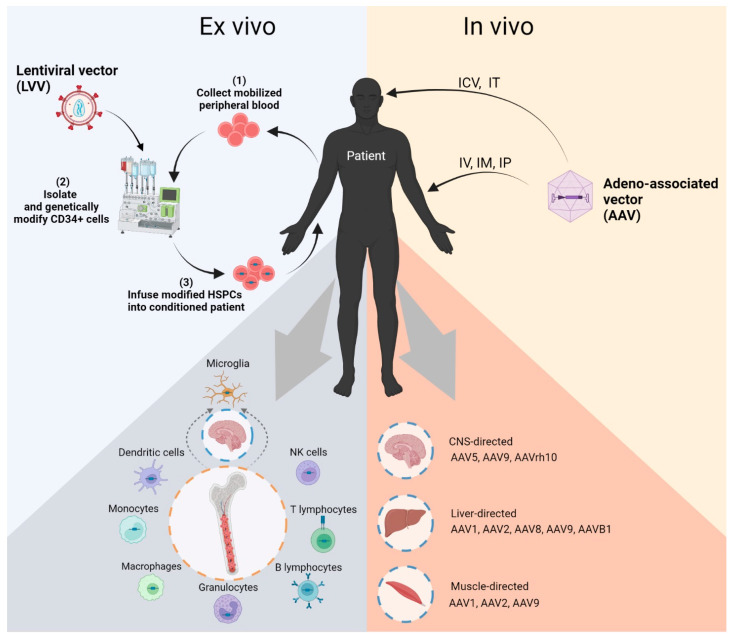
Overview schema of Pompe disease gene therapy modalities. Left panel represents the process of autologous ex vivo lentiviral gene therapy. Briefly, CD34+ cells are isolated in a closed manufacturing system from mobilized peripheral blood. These isolated cells are transduced with a lentiviral vector containing the functional gene of interest, and the genetically modified cells are infused back into a patient who has typically been conditioned with alkylating agents, such as busulfan, to create space in the bone marrow. Long-term repopulating stem cells engraft into the bone marrow niche and repopulate the hematopoietic system with cells capable of secreting functional enzyme, leading to uptake and cross-correction of affected peripheral tissues. The conditioning agent, busulfan not only makes space in the bone marrow for CD34+ to permanently engraft, but also enables the microglia in the CNS to be exchanged for gene-modified microglia derived from the infused cells. The right panel depicts in vivo AAV gene therapy approaches. Utilization of different AAV vector capsid proteins and routes of administration are used to target distinct tissue niches. Transduced cells secrete functional enzyme locally, or systemically depending on targeted tissue. ICV = intracerebroventricular, IT = intrathecal, IV = intravenous, IM = intramuscular, IP = intraperitoneal.

The predominant gene-transfer delivery systems investigated in preclinical and clinical settings are viral-based vectors designed to be utilized in in vivo and ex vivo gene therapies, which in the case of Pompe disease, is mainly centered around adeno-associated viral (AAV) vectors for in vivo applications and lentiviral (LV) vectors for ex vivo hematopoietic stem cell gene therapy. An overview of current gene therapy modalities for Pompe disease are presented in Figure 2.

### 3.1. AAV Vector-Mediated Gene Therapy in Pompe Disease

Gene therapy treatments for Pompe disease have been mostly tested in *Gaa*^−/−^ mice, a commonly used mouse model of Pompe disease [29,30]. One of the first gene therapy approaches to treat *Gaa*^−/−^ mice was using an adenoviral vector containing the CMV enhancer/promoter driving GAA cDNA expression, which resulted in systemic reduction of accumulated glycogen levels. In this experiment, one single IV administration was used to harness the capacity of the liver to secrete functional human GAA precursor enzyme, leading to high levels of GAA activity in the plasma, with subsequent cross correction and glycogen reduction in heart, skeletal muscle and smooth muscle [31]. Another approach used hybrid adenoviral-AAV vectors in preclinical studies, in which the properties of adenoviral (Ad) helper virus and AAV packaging were combined. These Ad-AAV-hGAA vectors produced detectable GAA protein after liver-directed targeting and were able to clear glycogen in the skeletal muscle of GAA KO mice [32,33]. Since then, the focus has primarily been on using AAV gene therapy for treating Pompe disease. AAV vectors contain an icosahedral capsid, and to date, multiple capsid serotypes have been characterized, each providing distinct tropism to cells found in the liver, muscle and CNS tissues. The receptors or coreceptors utilized by AAV for cellular entry are capsid serotype-specific and vary in abundance based on the cell type, which can be exploited to target disease-specific tissues [34]. These capsid serotypes can also be further modified to target novel tissues of interest. AAV contains a linear single-stranded DNA genome of about 4.7 Kb [35], and by removing the rep/cap genes, enough space is created to incorporate the human GAA cDNA (CCDS database: CCDS32760.1) of 2859 nt (952 amino acids) flanked by the inverted terminal repeats (ITRs). This *GAA* cDNA sequence can be driven by promoter elements to provide tissue-specific expression. Natural infection of AAV will not cause any human illness but the timing of the infection determines the immunogenicity [36]. The advantage of AAV vectors is the efficient transduction of terminally differentiated cells driving long-term expression, while remaining predominantly episomal, reducing the risk of insertional mutagenesis. However, AAV vectors have been reported to have the ability to integrate into the host genome, with the most common genomic integration site of AAV2 wild-type virus being AAVS1 [37]. This capability is thought to be lost when modifying AAV into gene therapy vectors, since the AAV Rep proteins essential for AAVS1 integration are absent [38]. More recently, clonal expansion of transduced cells was observed in hemophilia A dogs that were followed for about 10 years [39]. There were AAV integrations found in the genomic DNA of clonal dominant cells, thus emphasizing the need for long term follow up for potential genotoxicity associated with AAV vectors.

#### 3.1.1. Muscle Directed Delivery

Gene therapy targeting muscle could directly correct the affected tissue pathology involved in Pompe disease, and to that end, several AAV vectors with muscle tropism have been tested in preclinical and clinical studies. A recombinant AAV vector coding for mouse *Gaa* cDNA and packaged with recombinant AAV1 (rAAV1) serotype (AAV1/2) was effective in providing higher enzyme levels and improved reduction of glycogen deposits through intramyocardial and intramuscular injection into *Gaa*^−/−^ mice [40]. These results demonstrated the first attempts to resolve the cardiomyopathy seen in GAA knockout mice and restore the contractile functions of skeletal muscles using rAAV1/2 vectors. In addition, respiratory failure has been a typical hallmark in Pompe disease patients [41,42], and hence diaphragmatic delivery has been investigated. Consequently, intramuscular diaphragm delivery with AAV1 was attempted to improve respiratory functions. This method utilized a glycerin-based gel to deliver the vector within the diaphragm [43], and clearly demonstrated the tropism of AAV1 serotype in achieving uniform expression of GAA across the diaphragm. This AAV1-CMV-GAA vector driving GAA expression by the CMV promoter was used in preclinical studies in *Gaa*^−/−^ mice [44], and biodistribution/toxicology studies performed in New Zealand rabbits, showed diffusion of the vector throughout the diaphragm after localized injections [45]. The results showed significant improvement in respiratory functions when administered at an early age, but the effect declined with age. Interestingly, they have observed attenuation of efferent phrenic nerve activity indicating a retrograde transduction which warrants further details to study glycogen clearance from the peripheral nervous system. It has been reported that the use of the CMV promoter to drive transgene expression in viral vectors systems has been prone to silencing [46], which could explain the declining effect in these studies. The rAAV1/2-CMV-GAA vector was also administered intravenously in one-day-old mice [47]. This approach showed correction of muscle function and improvement of respiratory and cardiac functions, but a transient humoral response was seen 4–18 weeks following treatment. Furthermore, the vector AAV1-CMV-GAA was assessed for safety in diaphragmatic gene delivery in IOPD patients [48],(NCT 00976352). This clinical trial aimed to achieve higher respiratory performance compared to baseline and also anticipated to improve phrenic neuromuscular performance. The subjects enrolled showed significant improvements until day 180 but by day 365, the respiratory parameters started declining. Although the results were encouraging, there may still be a need for redosing to sustain GAA expression.

In addition to these studies, AAV vectors encoding human *GAA* expressed by the muscle creatinine kinase promoter was investigated in GAA-KO mice [49]. In these experiments, intramuscular injection of the vector was given to the mice resulting in the successful reduction of glycogen in skeletal muscles, however CNS correction was not reported. In this case, the presence of neutralizing antibodies to hGAA prevented the cross correction of cardiac muscle. This report also indicated that the choice of promoter showed dependency on AAV serotype and the muscle creatine kinase (MCK) promoter was active in heart with AAV2/7 serotype but not with AAV2/6 serotype. The neurological manifestations still remained a hurdle to complete normalization of the respiratory dysfunction associated with CNS glycogen accumulation.

In another preclinical study in Pompe mice, correction was shown in cardiac and muscular pathology with a construct containing a desmin (DES) promoter and a codon-optimized GAA sequence (rAAV9-DES-hGAA). This approach provided improvement in cardiac, skeletal and respiratory neuronal functions in Pompe mice, but the expression in *Gaa*^−/−^ mice was transient [50]. The desmin promoter exhibited more tissue-specific expression in skeletal muscles, cardiac muscle and motor neurons [51] and may reduce immunogenicity compared to using vectors with constitutive active promoters, e.g., CMV or chicken β-actin enhancer hybrid promoters to drive transgene expression. This study utilized rAAV9-DES-hGAA for systemic injection into *Gaa*^−/−^ mice and compared their results with ERT. The improvement in cardiomyopathy was not immediate but was observed three months post-injection. Immunogenicity modulation in response to GAA was significantly reduced compared to ERT. In a subsequent clinical study in LOPD patients, intramuscular delivery of AAV9-DES-hGAA in tibialis anterior muscle (NCT02240407) along with immunomodulation strategy to ablate B cells was tested to allow redosing with same vector to enhance the potential of the vector to correct the disease pathology in cardiac and skeletal muscles [52].

In a recent study in *Gaa*^−/−^ mice, as well as in non-human primates, systemic administration of the drug AT845, an AAV8 serotype vector containing the murine MCK promoter/enhancer driving codon-optimized human GAA resulted in supraphysiological enzyme activity levels leading to significant functional improvements and glycogen clearance in key target tissues in a dose-dependent manner. Although well tolerated at lower doses, high doses in cynomolgus macaques resulted in vector-related immune responses and inflammation, with cardiac abnormalities that necessitated the unscheduled euthanasia of two animals from the study. It was later concluded that the immune responses were in large part due to a xenogenic anti-GAA immune response, as an identical vector carrying macaque-derived GAA did not lead to the same inflammatory and cardiac pathology [53].

#### 3.1.2. Liver Directed AAV Gene Therapy

The liver is an important organ that naturally produces serum proteins, and it is often used as a depot for the efficient production of recombinant proteins for gene therapy applications. It also exhibits immune tolerizing properties aiding in prevention of transgene product-related immune responses if the introduced gene is specifically expressed in hepatocytes [54]. A liver-specific promoter (LSP) derived from the thyroid hormone–binding globulin promoter and a1 microglobulin/bikunin enhancer sequence was used in an AAV8-hGAA vector and given at a low dose in *Gaa*^−/−^ mice was effective at inducing immune tolerance induction in immunocompetent *Gaa*^−/−^ mice [55,56]. In addition, this liver-specific promoter resulted in effective glycogen reduction in heart and diaphragm when evaluated at 12 weeks after gene therapy. By administering low doses of the vector at 6 weeks before challenging the mice with rhGAA showed absence of antibody development, only when a hepatocyte-specific promoter was used, and the use of AAV2/8-LSP-hGAA could therefore enhance the efficacy of ERT in CRIM negative Pompe patients [57]. Furthermore, the efficacy of the construct AAV2/8-LSP-hGAA was tested for a medium effective dose in Pompe mice, in which the researchers found that the vector dose was inversely proportional to the production of anti-GAA antibodies [58]. These preclinical studies provided the rationale for an ongoing prospective, open-label trial in LOPD patients (NCT 03533673). In another approach, a hybrid tandem liver-muscle promoter (LiMP) using AAV8 and AAV9 serotypes was used in neonatal injections in *Gaa*^−/−^ mice, and this provided high and persistent multi-systemic transgene expression in non-dividing extra-hepatic tissues, and also aided in the prevention of anti-transgene immunity [59].

**Figure 3:**
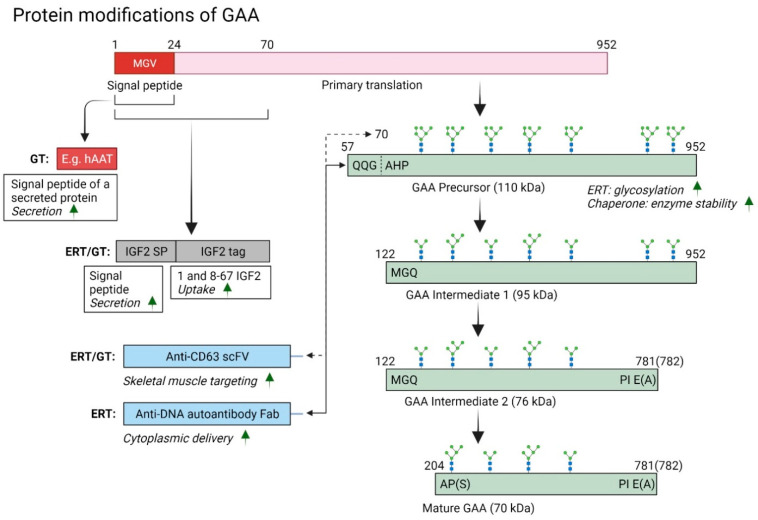
Summary of GAA protein modifications employed in ERT and gene therapy. A redrawn and modified model for maturation of GAA protein, previously reported by Moreland et al. [13]. Modifications to GAA transgene or protein described in literature were subject to changing the N-terminus of the GAA protein to improve secretion and uptake in the key tissues affected in Pompe disease without affecting proteolytic processing steps to the mature GAA protein. Signal peptides have been modified to improve secretion, and tags have been incorporated to enhance uptake in skeletal muscles or cellular delivery to cytoplasm to degrade glycogen more effectively. For enzyme replacement therapy enhanced glycosylation or chaperone has been investigated as well. GT = gene therapy, ERT = enzyme replacement therapy, Fab = antigen-binding fragment. scFv = single-chain variable fragment, hAAT = human alpha-1-antitrypsin.

However, although the liver is efficient in secreting recombinant proteins, GAA protein is generally poorly secreted from transduced cells [60]. Hence, several GAA protein modifications have been investigated to optimize transgene expression and enhance secretion of GAA protein (Figure 3). Sun et al., studied whether the signal peptide of the GAA sequence could be replaced by the signal peptide of a secreted protein [61]. Five signal peptides were tested in cell lines, and replacing the GAA signal peptide with the signal peptide of human α1- antitrypsin (hAAT) resulted in enhanced GAA protein secretion and also showed improved efficacy in immunocompetent *Gaa*^−/−^ mice. Another strategy investigated liver tropic rAAV8 delivery resulting in low immunogenicity and partial cross correction of CNS and muscle pathology [60,62]. In this study, different secretable signal peptides and a deletion of eight amino acids at the N terminus fused to codon-optimized GAA, was used to enhance GAA protein secretion and reduce immunogenicity. An AAV8 serotype with a liver-specific apolipoprotein control region and alpha-1-antitrypsin promoter (hAAT) that was previously used for liver-directed gene therapy of Crigler Najjar syndrome [63] showed that the construct AAV8-hAAT-sp7-delta8-coGAA was very efficient in muscle glycogen clearance and reduced pathology, but glycogen reduction in CNS was less prominent [60]. However, brain and spinal cord sections showed the presence of lysosomal GAA and reduction in Iba1+ cells indicating an effect on neuropathology. The high amount of secreted GAA from the liver in non-human primates and reduced anti-GAA antibodies to the sp7-delta8-coGAA confirmed reduced immunogenicity with increased GAA activity in plasma and skeletal muscles. In a follow-up study, AAV8 secretable GAA was compared to ERT in *Gaa*^−/−^*Cd4*^−/−^ immune-compromised mice, and liver-directed gene therapy, and particularly at high dose (2 × 10^12^ vg/kg) provided robust GAA activity in muscles and glycogen reduction [28]. In the CNS, GAA protein and activity were significantly increased in the spinal cord and brain, but effects on reducing glycogen in brain were less pronounced compared to the spinal cord.

An investigational clinical trial using a rAAV with bioengineered Rh74-derived capsid and an expression cassette to drive the expression of secretable GAA protein (SPK-3006) is currently being sponsored by Spark Therapeutics (NCT04093349). A recently published preclinical AAV gene therapy study by Baik and colleagues [64], the catalytic portions of GAA were fused to a single-chain variable fragment (scFv) directed to CD63. The antigen CD63 was selected by a screening performed to select effector proteins that traffic from the cell surface to the lysosome, and which are expressed on skeletal muscle but minimally expressed in the liver. This approach was tested as an ERT, but also through AAV8 liver-specific expression, and showed superiority in reducing glycogen, and reduction in autophagic build-up in skeletal muscles and subsequent improvement of muscle function.

#### 3.1.3. CNS Directed AAV Gene Therapy

CNS manifestations in Pompe disease are prominent and are an important component that needs to be addressed in a gene therapy approach for both IOPD and LOPD patients [65,66]. In order to prevent, halt or potentially reverse the CNS pathology several attempts with recombinant AAV vectors were carried out. An AAV5 serotyped AAV vector encoding GAA protein, AAV5-GAA was injected at the C3-C4 spinal level of adult GAA KO mice resulting in attenuation of glycogen in the cervical ventral horn along with the spinal cord, and subsequently showed potential respiratory function improvement [67]. This emphasizes that the therapy needs to be targeted to both the CNS and skeletal muscles to effectively treat Pompe disease. Several preclinical studies were later performed with different AAV serotypes attempting to evade the immune response and improve efficacy.

The AAV9 serotype has shown effective CNS tropism in neonatal and adult mice [68], but also targets liver and skeletal muscle [69]. The neuronal tropism of AAV9 has been exploited for gene therapy application of spinal muscular dystrophy expressing the survival motor neuron (*SMN*) gene by the CMV enhancer/chicken β-actin hybrid (CAG) promoter (NCT02122952) [70,71], which resulted in the recent approval of onasemnogene abeparvovec (Zolgensma). Using the AAV9 serotype, Hordeaux et al. [72] used single intrathecal delivery of AAV9-CAG-hGAA in *Gaa*^−/−^ mice and demonstrated neurological correction and associated improvement in cardiac functions.

Glycogen accumulation in Pompe disease occurs in motor neurons that contribute to neuromuscular dysfunction [5,60]. Hence, neuron-specific synapsin I promoter gene delivery was attempted with a construct yfAAV9/3-Syn-I-hGAA [73]. In this study, neonatal *Gaa*^−/−^ mice were injected intracerebroventricularly resulting in efficient expression of GAA in neuronal cells, predominantly in the cortex and cerebellum. Glycogen deposits were not eliminated in the liver and quadriceps, but the brain and spinal cord had significant reduction as well as a mitigation of astrogliosis. There was a slight improvement in muscle strength, but locomotor function assessed by rotarod was normalized. Another approach investigated AAV-B1, a serotype that was isolated from a novel CNS tropic AAV capsid, after a single round of in vivo selection from an AAV capsid library [74]. AAV-B1 effectively transduced CNS and muscle cells, and thereafter was tested in three-month-old *Gaa*^−/−^ mice using a desmin promoter to drive *GAA* expression for muscle and CNS correction [75]. This vector efficiently transduced the tongue and also showed improvement in respiratory pathology and increased grip strength, while cardiac transduction cleared glycogen in the myocardium with moderate transduction observed in motor neurons. The results using the novel AAV-B1 serotype were similar to a control AAV9 vector.

In another study, using an intralingual route of injection of recombinant AAV1 and AAV9 vectors in *Gaa*^−/−^ mice, resulted in efficient transduction of tongue fibers, but AAV9 was more efficient in transducing motor neurons [76]. To enhance the muscular efficacy of the therapeutic protein, GILT-tagged GAA with a portion of IGF2 fused to GAA was developed. This GILT-tagged GAA was previously investigated for ERT in Pompe mice, and demonstrated to be fivefold more effective than equivalent doses of rhGAA at clearing glycogen in skeletal muscle [77] as well as improved respiratory function in a Pompe mouse model [78]. In other LSD models, e.g., MPS VII mice, using IGF2-tagged proteins for ERT was also able to more effectively reduce storage product in key tissues [77,79]. Recombinant GILT-tagged GAA was also investigated in a phase 1/2 clinical trial (NCT01230801) [80], in which patients showed initial improved respiratory measures as well as a limited increase in walking endurance. However, the GILT tagged GAA (reveglucosidase alfa) induced transient mild hypoglycemia in a few patients due to the IGF2 moiety that can bind the insulin receptor with low affinity. Hypoglycemia was particularly observed in the high-dose groups receiving 20 mg/kg. This IGF2-tagged GAA was incorporated in a AAV9-DES-IGFIIcoGAA vector, and after intralingual injection of IGF2-tagged GAA protein was present in XII motor neurons as demonstrated by immunohistochemical staining of GAA, increased GAA activity in tongue lysates and enhanced reduction of glycogen accumulation [81]. Another optimized gene therapy vector developed by Amicus Therapeutics/University of Pennsylvania using a pan-tropic AAV capsid carrying an engineered GAA transgene modified for improved secretion and uptake has been communicated, which efficiently corrected heart, CNS and muscle pathology after IV administration of 2.5 × 10^13^ vector genomes/kg in young *Gaa*^−/−^ mice [82,83,84]. Finally, novel AAV-capsid from Abeona Therapeutics’ next-generation AIM™ AAV vector platform has been reported to have improved biodistribution in heart, muscle and CNS, and is now in preclinical development for Pompe disease [85].

Several modifications to AAV serotypes have been used in gene therapy trials with enhanced safety and long-term efficacy across target organs to correct the pathophysiology across the CNS, heart, and skeletal muscles. There were also several successful preclinical attempts with these recombinant AAV vectors secreting high amounts of GAA and able to directly correct CNS resolving the respiratory dysfunction in Pompe disease mice. Although clinical trials assessing safety show promising results, the ability to mount immune responses to the capsid protein and transgene product, as well as pre-existing neutralizing antibodies against AAV might compromise safety and clinical efficacy. AAV vector infusions can cause inflammatory toxicities that increase with vector dosing, complement activation, cytopenias and marked hepatotoxicity [86]. Additionally, infusing high doses of 2 × 10^14^ genome copies per kilogram bodyweight of an AAV9 variant systemically in non-human primates and piglets, caused liver and sensory neuron toxicities independent of an immune response to the capsid or transgene product [87]. Furthermore, in patients with X-linked myotubular myopathy, the investigational therapeutic *MTM1* (myotubularin) named AT132 using an AAV8 resulted in four deaths showing liver dysfunction from 3–4 weeks after receiving AT132. All patients had evidence of pre-existing intrahepatic cholestasis, implicating disease background may have played a role, but more investigation is required [88].

Prior exposure to wild-type (WT) AAV by natural infection develops humoral immunity in humans against the capsid protein [89]. The anti-AAV neutralizing antibodies and the cross-reactive antibodies across various AAV serotypes blocks target cell transduction and thereby significantly alter the therapeutic efficacy of the administered rAAV with the transgene [90,91]. These rAAV capsids and the transgene product have the ability to activate both humoral and cellular responses contributing to activating cytotoxic T lymphocyte (CTL) responses and complement proteins [92]. These innate problems associated with rAAV need to be carefully studied in order to improve overall outcomes. The pivotal role of TLR9 activation [93] as a mediator of both the cellular and humoral immune response needs to be analyzed further, as well as CpG motifs in the vector genome, which can drive the immune responses associated with CD8+ T-cell activation [94]. The introduction of novel technologies, such as incorporating short DNA oligonucleotides in vector genomes that antagonize TLR9 activation, may be beneficial in reducing TLR9-mediated immune responses and may allow higher doses to be infused in patients [95].

### 3.2. Lentiviral Mediated HSPC Gene Therapy in Lysosomal Storage Disorders

Gammaretroviral and lentiviral vectors have been used in hematopoietic stem cell gene therapy for both blood disorders and lysosomal storage disorders (LSDs). In HSPC gene therapy trials for X-linked severe combined immunodeficiency and Wiskott–Aldrich syndrome using early design gammaretroviral vectors, which resulted in T acute lymphoblastic leukemia in a considerable proportion of patients [96]. In the Wiskott–Aldrich syndrome clinical trial, nine out of 10 treated patients had successful engraftment, but seven patients developed acute leukemia with integration site analysis revealing insertions near proto-oncogenes *LMO2*, *MDS1* or *MN1* [97]. In ADA-SCID, however, retroviral gene therapy did not show evidence of leukemic transformation, despite integration in *LMO2* and other proto-oncogenes [98,99,100,101], indicating that insertional oncogenesis is vector and disease-specific. In light of these early trials, the field has since moved away from gammaretroviral-based vectors and instead utilize safer third-generation self-inactivating (SIN) LV vectors as the platform for HSPC gene therapy.

LV vectors are able to efficiently transduce both dividing and non-dividing cells, e.g., neurons [102], but HSPCs require an additional cytokine stimulation step for efficient ex vivo transduction [103]. The current state-of-the-art LV vectors are third-generation SIN vectors, for which the packaging sequences and transfer vector are divided among four plasmids to further reduce the risk of recombination events [104,105,106]. The SIN configuration of the long-terminal repeat (LTR) improved safety and allowed controlled transgene expression by an internal promoter, while the addition of a Woodchuck hepatitis virus posttranscriptional regulatory element significantly improved transgene expression [107]. The most commonly used envelope glycoprotein for human HSPC LV transduction is the vesicular stomatitis virus g-protein (VSVg), which imparts broad cell tropism [108]. Pseudotyping LV vectors with VSVg also provides stability to the vector particles, which can readily be concentrated to high titers using ultracentrifugation [109]. Although VSVg is commonly used for LV vector pseudotyping [108], other glycoproteins, such as RD114/TR and baboon retrovirus envelopes, are capable of efficiently mediating transduction of human HSPCs, and may be a suitable, even beneficial, alternative to VSVg pseudotyping. Third-generation SIN LV vectors are now the predominant vector system used in HSPC gene therapy trials and exhibit improved efficiency and reduced genotoxicity profiles in preclinical and clinical settings [110].

LV HSPC gene therapy has been successfully applied in clinical trials for the treatment of metabolic diseases, such as metachromatic leukodystrophy (MLD) [111], Fabry disease [112] and Hurler disease [113], as well as for peroxisomal disorder X-linked cerebral adrenoleukodystrophy (CALD), with reported follow-ups of 12 years after transplantation [114,115,116]. Overall, more than 350 patients have been treated with lentiviral hematopoietic stem cell gene therapy for monogenic disorders without any serious genotoxic-related adverse events [117]. The benefit of using HSPCs relies on the ability of ex vivo transduced HSPCs to durably engraft in the bone marrow niche and give rise to the full hematopoietic cellular repertoire (including microglia in the CNS), which then act as factories providing local delivery of enzyme to disease target tissues, and systemic delivery through circulation in the plasma. Immune tolerance induction to recombinant therapeutic enzymes has been shown in allogeneic hematopoietic cell transplantation in Hurler’s disease [118], and in Fabry HSPC gene therapy [112], whereby antibodies against recombinant protein resolve over a few months, making LV HSPC gene therapy a platform with very low immunogenicity risk.

An additional benefit of LV HSPC gene therapy is the ability of modified HSPCs to differentiate into microglial cells and engraft into the CNS compartment [119]. LV gene therapy modified microglial cells can provide robust expression of protein throughout the CNS and may provide significant therapeutic benefit through cross-correction of disease-affected glial cells. This characteristic of HSPC gene therapy was successfully demonstrated in preclinical gene therapy studies in MLD mouse models, in which high levels of engraftment in bone marrow after busulfan conditioning was achieved with genetically modified HSPC microglia acting as sources of Arylsulfatase A enzyme production in the CNS, resulting in the amelioration of neurological deficits [120,121]. In MLD patients receiving allogeneic HCT, there was no evidence of cross-correction of oligodendrocytes and astroglia; however, donor macrophages were able to efficiently digest accumulated sulfatides, which may have played a direct neuroprotective role for resident oligodendrocytes, enabling remyelination [122]. These results highlight the ability of LV HSPC gene therapy to penetrate into the CNS niche and provide therapeutic benefits to LSDs with neurological pathology.

#### 3.2.1. Lentiviral Vector HSPC Gene Therapy for Pompe Disease Treatment

Ex vivo hematopoietic stem cell gene therapy has also been tested preclinically for the treatment of Pompe disease, with results showing robust systemic GAA expression and the ability to correct neurological deficits in Pompe mouse models [123,124,125]. Transplantation of syngeneic genetically modified HSPCs has the advantage of inducing immune tolerance to endogenously secreted proteins and infused recombinant proteins as previously reported in several preclinical models [123,126,127]. This approach could provide long-lasting therapeutic benefit through the single administration of transduced cells, and can potentially be used to treat both IOPD and LOPD patients, assuming sufficiently high levels of GAA expression beyond the minimum disease threshold are achieved. The first preclinical study that reported HSPC gene therapy for Pompe disease was by Douillard-Guilloux et al. [123]. Here the authors used third-generation SIN LV vectors expressing human GAA driven by a ubiquitous MND (myeloproliferative sarcoma virus enhancer, negative control region deleted D1857 rev primer binding site substituted) promoter to transduce HSPCs before infusing into irradiated *Gaa*^−/−^ mice. Results showed low but detectable GAA enzyme activity in peripheral blood and bone marrow cells (~50–80% of wild-type levels) at 17 weeks after infusion, and chimerism determined in colony-forming cells was approximately 13–20%. Despite low levels of GAA enzyme, significant glycogen reduction was observed in gastrocnemius tissue, although not in the heart, and results were comparable to administration of rhGAA alone. Importantly, in HPSC gene therapy-treated *Gaa*^−/−^ mice, IgG immune responses are undetectable through immune tolerance induction against the transgene product—one of the key benefits of the tolerogenic nature of HSPC transplantation [123].

In another preclinical study, a vector containing the strong spleen focus forming virus (SFFV) promoter was used to drive the expression of the *GAA* transgene in hematopoietic cells [124]. Supraphysiological levels of GAA enzyme activity were achieved at eight months after infusion of genetically modified cells, reaching ~13–48-fold higher levels in peripheral blood and spleen compared to wild-type controls. Bone marrow chimerism was roughly ~35% with an average VCN per diploid genome of 7.3. GAA enzyme activity persisted up to 18 months after transplantation, and there was a significant reduction in glycogen deposits in the heart, diaphragm, lung, liver and spleen, without a reduction in glycogen in the brain. Functional improvements in locomotor and respiratory function were observed, as well as reversal of cardiac remodeling. Overall, this attempt was successful in restoring partial functions of the affected organs and tissues but did not achieve biochemical correction in the CNS.

**Figure 4:**
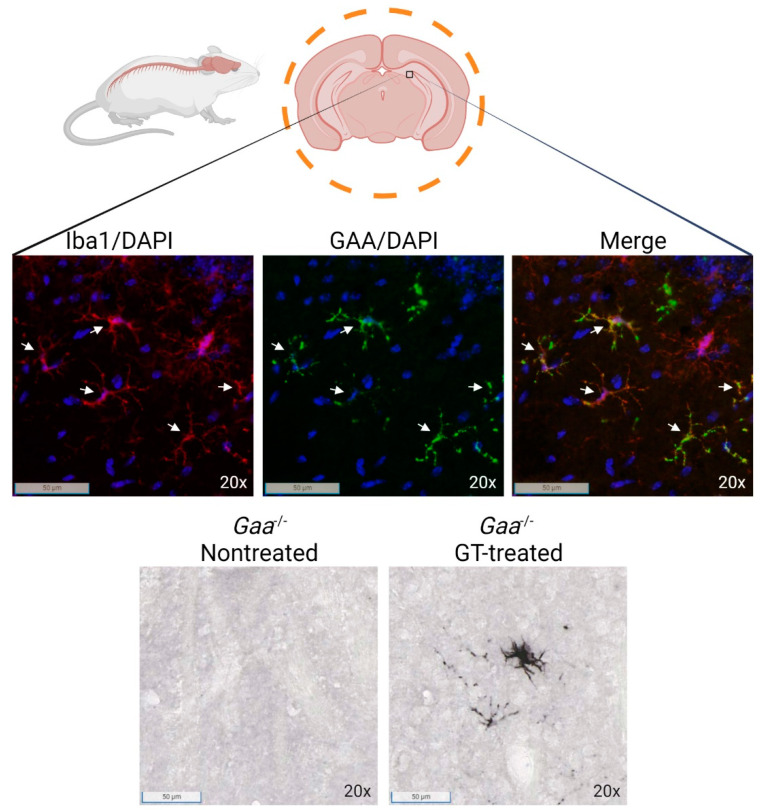
GAA protein expression in resting microglia in the brain of LV HSPC gene-therapy treated *Gaa*^−/−^ mice. Genetically modified *Gaa*^−/−^ lineage negative bone marrow cells transduced with a lentiviral vector with codon-optimized *GAA*, driven by the spleen focus forming virus (SFFV) promoter were infused in to *Gaa*^−/−^ mice [29] after busulfex conditioning. Brains were harvested at six months after infusion of genetically modified cells in *Gaa*^−/−^ mice, and post saline perfusion fixed in 4% formaldehyde for 24 h, and subsequently processed for immunofluorescence and immunohistochemical staining for GAA protein. Top panels: Representative anti-GAA (green) and Iba1 (red) immunofluorescence staining shows GAA colocalization in engrafted microglia-like cells in the hippocampal region. DAPI is shown in blue. Bottom panels: representative images of immunohistochemical staining for GAA. White arrows indicate microglia cells colocalizing with GAA signal.

Another modification to the LV vector expression cassette was to incorporate a codon-optimized *GAA* transgene (GAAco) to improve overall expression [125]. The subsequent preclinical study demonstrated improved GAA protein production in circulation compared to the native *GAA* sequence, which cleared the glycogen deposits from the heart and skeletal muscles and enabled improved locomotor function and resolution of cardiac hypertrophy at a ~7 VCN/dg. Importantly, the human GAA protein was also detected in microglia and astrocytes of transplanted mice. The presence of genetically modified microglia throughout the brain in transplanted *Gaa*^−/−^ mice can clearly be demonstrated (Figure 4), and GAA protein detected in astrocytes suggests efficient cross-correction [125]. Even with the high average VCN, integration site analysis showed an expected lentiviral pattern, with no proto-oncogene selection and without any genotoxicity-related adverse effects. Nonetheless, VCN/dg were generally higher than the FDA-guided maximum vector copy number, which is <5 copies per diploid genome [128]. Higher chimerism of genetically modified cells may allow for improved glycogen clearance at a lower average VCN than what was obtained in the studies by Douillard-Guilloux [123], van Til [124] and Stok et al. [125].

Although the viral SFFV promoter has been used in a clinical trial for ADA-SCID [129], less-strong human promoters for clinical application are preferred. Hence, the elongation factor 1 alpha short promoter (EFS) in combination with elements from the β-globin locus (LCR-EFS) has been explored for ADA-SCID gene therapy to enhance expression in erythrocyte progenitors [130]. It was postulated that using this LCR-EFS enhancer/promoter fusion promoter could also be beneficial in the treatment of LSDs. In a more recent publication, an LV vector with the erythroid-specific LCR-EFS element driving *hGAA* cDNA partially rescued clinical manifestations in a murine Pompe disease model [131]. GAA expression was 3–6-fold preferentially higher in erythroid cells, which enhanced GAA secretion. In *Gaa^−/−^* mice, the biochemical correction was observed in heart, but not in skeletal muscle, lung and brain. Consequently, heart parameters were improved, i.e., reduction in heart mass after treatment, but with no improvement in rotarod or grip strength measurements. The VCN was relatively low in this study (~0.6 VCN/dg in blood with a range 0.36–1.44 VCN/dg) at 36–100% donor cell chimerism, and it may be advantageous to increase the average VCN for improved biochemical correction. Other constitutively active promoters and/or lineage-restricted promoters to drive robust *GAA* expression in hematopoietic lineages may provide a more favorable risk/benefit profile. Of note, human CD34+ HSPCs were efficiently transduced without losing their stemness or differentiation potential, and successfully expressed GAA in plasma of the transplanted NSG (NOD.Cg-*Prkdc^scid^Il2rg*^*tm1Wjl*^/SzJ) mice [131].

To enhance delivery to target tissues, in vitro studies showed that IGF2-tagged GAAco improved secretion and uptake in a transwell system [132]. To that effect, a lentiviral vector using the MND promoter driving expression of a IGF2-tagged GAA was used in LV HSPC gene therapy. In this study, nine GAA chimeric variants driven by the MND promoter were investigated [133], including tags described to enhance delivery to the CNS, such as an apolipoprotein E (ApoE) tag [127] and a modified IGF2 tag containing a R37A mutation for GILT delivery [134]. The MND-GILT-R37A-GAAco vector was more effective in reducing glycogen in skeletal muscles and CNS than the non-tagged GAAco vector 16 weeks after transplantation of genetically modified HSPCs. Effects in the CNS were found to be achieved from a relatively low percent of 0.1–3.7% microglial cell engraftment in the brain, approximated using surrogate control *Gaa*^−/−^ mice transplanted with HSPCs transduced with a GFP vector.

Using a *hGAA* transgene, CNS penetration and correction were minimal or only achievable with very high systemic expression levels. Pre-transplant conditioning plays an important role in the successful engraftment of the transduced HSPCs in bone marrow and CNS [135]. For this to be more efficient, the conditioning regimen should be able to ablate resident microglial cells and increase the turnover of donor-derived microglial cells as local producers of the therapeutic protein. The conditioning regimens commonly used in preclinical animal models are irradiation and chemotherapeutic agents, such as the alkylating agent busulfan (1,4-butanediol dimethanesulfonate). Since microglia account for ~5–12% of cells in the rodent brain [136], the therapeutic enzyme levels depend on the percentage of microglial engraftment in the brain, the transgene promoter activity in microglial cells, and the efficiency of cross-correction. The GAA enzyme activity levels in the CNS of treated Pompe mice were lower than the activity level observed in wild-type animals [133]. However, the low enzyme activity levels were similar to those seen in other studies using HSPC gene therapy, such as *Arsa*^−/−^ mice reaching close to 10% of wild-type arylsulfatase A enzyme activities [121], and *Ids^−/−^* treated mice which did not exceed 4% of wild-type levels in the brain. Interestingly, heparan sulfate and dermatan sulfate levels were completely cleared even at these levels of enzyme activity [127].

An exception to these enzyme levels was observed in gene therapy-treated *Idua^−/−^* mice, which resulted in 4.5-fold wild-type levels [137]. Using irradiation or busulfan conditioning, the number of gene-modified microglia cells in the brain was approximately in the range of 10% of the microglia cells in the brain, which is around 1% of total CNS cells. Depending on how much therapeutic protein is required to achieve efficient cross-correction, it could be advantageous to increase the proportion of gene-modified microglia in the CNS. Colony-stimulating factor (CSF1R) is essential for microglia survival [138] and its receptor tyrosine kinase activity can be inhibited by the brain penetrant chemical PLX5622. PLX5622 depletes endogenous microglia and its withdrawal resulted in repopulation of the microglial cell niche from the remaining residual microglial cells [139], demonstrated in mouse bone marrow transplantation settings, in which PLX5622 treated mice were subjected to 9 Gy whole-body irradiation followed by whole bone marrow cell transplantation [140]. After 30 days ~93% of the microglial cells in the brain were donor-derived (~99% in retina and ~93% in spinal cord). This experiment revealed the potential advantage of using CSF1R inhibitors to increase the number of donor-derived cells in the brain, which is an important parameter in treating diseases with CNS pathology. Further studies are needed to investigate the safety of this drug, but ongoing studies testing the safety of CSF1R targeting agents may provide compounds to increase the engraftment of genetically modified cells in the CNS [141,142].

Another approach could be to use antibody fusion proteins to either neonatal Fc receptor, a low-density lipoprotein receptor-related protein, the transferrin receptor (TfR), or the insulin receptor in a gene therapy setting to enhance delivery directly through the BBB into the CNS, as was done previously in attempts to improve ERTs [24]. However, targeting the insulin receptor, as well as other receptors, may come with the risk of transient hypoglycemia and other unwanted side effects [143].

#### 3.2.2. In Vivo Lentiviral Gene Therapy for Pompe Disease

In addition to ex vivo Lentiviral gene therapy approaches, direct in vivo applications have also been explored for the treatment of Pompe disease. Testing this modality in vivo relies on taking advantage of the relative immature immune systems in newborn mice [144]. In line with this strategy, Kyosen et al., used a LV vector encoding GAA driven by the CMV promoter and IV injected it directly into neonatal mice [145]. The GAA protein detected in plasma stabilized from +/− 8 weeks and was followed up to 24 weeks. The results indicated effective glycogen reduction in the heart but not in the diaphragm and quadriceps. The neonatal delivery has the advantage of minimizing immune responses, as observed by lack of CD4+ and CD8+ cell infiltrates in tissues, with few mice developing anti-GAA antibodies. However, further improvements are needed to efficiently clear glycogen deposits from cardiac, respiratory, skeletal muscle and CNS tissue. Unfortunately, in vivo lentiviral gene therapy for Pompe disease has only achieved low efficacy, a platform perhaps more suitable for diseases that do not require such high protein levels as is required in Pompe disease. Lentiviral gene therapy has been investigated in large animal preclinical models for hemophilia in dogs [146] in which VSVg pseudotyped lentiviral vectors with an enhanced transthyretin hepatocyte-specific promoter driving codon-optimized hyperfunctional canine Factor IX carrying the R338L mutation, and four tandem repeats of miR-142 target sequences to limit expression in antigen-presenting cells were used. After intrahepatic portal vein delivery, this approach reached 1% of normal FIX activity, but manufacturing capacity and observed infusion-related complications may limit applications for lentiviral-directed gene therapy in the context of Pompe disease. More recent modifications on lentiviral vector producer cells to alter the lentiviral particle composition by removing human leukocyte antigens and incorporating ‘do-not-eat-me’ signals to prevent phagocytosis by Kupffer cells improved liver transduction significantly [147]. Unfortunately, this approach of liver-directed targeting would fail to efficiently target the CNS compartment, which would be needed to address CNS associated disease pathology seen in Pompe disease. Similar to this predicament, in targeting the CNS compartment, as has been shown in the non-human primate Krabbe models which demonstrated efficient lentiviral vector transduction of neurons, astrocytes, and oligodendrocytes near the site of injection, one would fail to target key tissues in the periphery [148].

### 3.3. Alternative Applications to Modulate GAA Mutations and Disease Correction

In addition to cell and gene therapy approaches, alternative molecular-based strategies are being explored for the treatment of PD. For instance, chemically modified antisense oligonucleotides (AON) insensitive to RNase-mediated degradation have been tested in preclinical settings to enhance endogenous production of wild-type GAA enzymes [149,150]. Van der Wal et al. identified overlapping AONs that increased GAA activity in patient-derived cells carrying a commonly reported splice variant above disease thresholds by blocking a negative splicing element, resulting in the inclusion of exon 2. These findings provide proof of concept for using this experimental modality to correct aberrant splicing and mediate exon inclusion. Importantly, AON treatments have been well-tolerated in clinical trials for spinal muscular atrophy and Duchenne muscular dystrophy [151,152]. However, because AONs are sequence-specific, they are usually only suitable for a small subgroup of patients with similar gene variants, which limits a use case for a broader patient population. This therapeutic approach would also fail to provide the supraphysiological expression likely needed to prevent or reverse the most severe forms of PD. However, alternate modalities of therapy in conjunction with more potent forms of therapy could potentially benefit PD patients.

Targeting satellite cell activation in Pompe disease is another active area of research that could potentially be used in combination with the above-mentioned gene therapies. Skeletal muscle can regenerate in response to damage by recruiting the activity of tissue-resident stem cells. Skeletal muscle stem cells (MuSCs), also known as muscle satellite cells, rapidly activate after sensing tissue damage and proliferate to regenerate damaged myofibers. Although MuSC numbers have been shown to be stable in PD patient biopsies, Schaaf et al. found that MuSCs were lacking markers of active regeneration, namely the expression of embryonic myosin heavy chain [153]. Similarly, in Pompe mouse models, *Gaa*-deficient mice also show muscle wasting and an absence of MuSC activation and regeneration, despite ruling out cell-intrinsic defects [154]. The inability to efficiently activate MuSCs to restore muscle regeneration likely contributes, in part, to ongoing muscle wasting seen in Pompe patients and suggests that the surrounding niche may suppress MuSC activation. These findings provide a therapeutic rationale to investigate methods able to target MuSCs with the hopes of attenuating PD muscle pathology. Satellite cells have been shown to be safely and efficiently activated through physical exercise [155,156]. Previous exercise programs were found to be beneficial for adult Pompe patients and present a sensible supplemental therapy. Small molecules targeting and activating MuSCs or targeting indirectly through connected pathways, such as autophagic flux, is another possibility.

The use of protein engineering strategies has also shown promise in generating more stable GAA proteins, an application that would complement both ERT and gene therapy efforts. Using directed evolution and high-throughput screening methods, Dellas et al. and Botham et al. demonstrated that modified GAA amino acid sequences, containing up to 30 amino acid changes from the reference sequence, could drastically help increase protein stability at neutral and low pH ranges, increase expression and increase cellular uptake in Pompe disease fibroblasts and myoblasts [157,158]. These results will need to be corroborated in an in vivo setting to prove meaningful efficacy and safety of substituting a substantial number of amino acids; however, this strategy highlights another interesting avenue in which innovation could help leverage existing treatment modalities.

Recent advances in gene-editing techniques provide exciting new opportunities for the treatment of metabolic genetic disorders. Although in vivo targeting of non-dividing tissues remain challenging, novel base-editing approaches offer solutions to a subset of diseases whereby homologous-directed repair and double-stranded DNA breaks are unnecessary. Base editing allows genome editing independent of HDR and dsDNA breaks through a cytidine or adenosine deaminase fused to a catalytically inactive Cas9 [159]. Villiger et al. recently demonstrated the feasibility of this approach with the correction of phenylketonuria in a mouse model for the human autosomal recessive liver disease using CRISPR/Cas-associated base editors [160]. Using a novel intein-split base editor, Villiger and colleagues were able to bypass AAV cargo size limitations and deliver the fusion protein in two parts. Delivery of the AAV-base editor system resulted in physiological blood phenylalanine levels below 120 µmol/L with restoration of enzyme activity and reversion of the light fur phenotype.

In theory, genome editing in Pompe disease would be an attractive strategy, as it would permanently correct mutations in the GAA gene and restore enzyme function under normal physiological control. However, given that Pompe disease would require widespread biodistribution and extremely high efficiencies of correction in both muscle and CNS tissue to halt disease progression and reverse disease pathology, the application of this technology, especially using homology-directed repair, to correct mutations in Pompe disease is not currently feasible. Although base-editing approaches have shown efficacy in other diseases as mentioned above, using base editing to treat PD would be difficult due to the wide range of mutations seen in the *GAA* gene of the patient population, which would each require a unique guide RNA sequence. However, using CRISPR-based genome editing strategies could prove useful in ex vivo gene therapy settings, as it would assuage any safety concerns regarding integration associated genotoxicity related to retroviral vector-mediated gene delivery, although unwanted off-target mutations would still have to be ruled out.

Finally, the availability of CRISPR tools to generate novel mouse models that better recapitulate human disease phenotypes could help accelerate the design of the next generation of PD therapies. Huang et al. recently reported the generation of a novel murine IOPD model utilizing dual sgRNA CRISPR-Cas9 homology-directed recombination to harbor the orthologous *Gaa* mutation c.1826dupA (p.Y609 *) [161]. This model effectively recapitulated the patient-specific genotype, which results in hypertrophic cardiomyopathy and skeletal muscle weakness, hallmarks of human IOPD. This mouse model can be instrumental in testing gene-editing and base-editing strategies and could be leveraged to find more effective therapies.

## 4. Conclusions

ERT is the current standard of care for the treatment of both IOPD and LOPD, but unfortunately, while being a major advance in Pompe patient care, ERT still has considerable shortfalls, including the ability to impact the CNS. The advent of gene therapy holds great promise for the potential prevention, halting, and/or reversal of Pompe disease. When compared to other lysosomal storage diseases, Pompe disease requires larger quantities of functional recombinant human GAA protein distributed throughout the body, including the CNS. A potential solution is gene therapy. AAV vectors or LV HSPC mediated gene therapy may be able to provide improved bioavailability of the transgene product to enhance efficacy. Several factors for in vivo AAV gene therapy, such as the quantity of dosing, biodistribution, and immunogenicity to the vector and transgene product, need to be thoroughly assessed, but technological advancements to the vector design and transgene may limit vector-associated immunogenicity risks and improve efficacy. Alternatively, LV HSPC gene therapy could provide the required systemic delivery, with optimizations in conditioning and transduction efficiency of HSPCs to maximize peripheral and CNS engraftment of genetically modified cells for optimal efficacy. The benefit-to-risk ratio associated with treatment modalities in which disease background could play a major role necessitates scrutinizing every crucial step in the preclinical development. Gene therapies have the potential to be single-dose treatments, enabling lifelong therapeutic GAA expression with the possibility of preventing, halting or reversing Pompe disease.

## Figures and Tables

**Table 1:** Overview of academic and company-sponsored preclinical, clinical stage and marketed therapies to treat Pompe disease. Gene therapy applications are in the transitional development stage to clinical application. Clinicaltrial.gov numbers are provided in bold. Ab = antibody, AAV = adeno-associated viral, CMV = cytomegalovirus enhancer/promoter, HSPC = hematopoietic stem and progenitor cell, LV = lentiviral vector, ERT = enzyme replacement therapy, IOPD = infantile-onset Pompe disease, LOPD = late-onset Pompe disease. Yellow = ERT product; Gray = chaperone therapy; Orange = AAV Gene Therapy; Blue = HSPC LV Gene Therapy.

Preclinical Program	Clinical Stage	Marketed Authorization
AAV2/8 Gene Therapy delivered Ab-GAA (Regeneron)	AAV2/8 LSPhGAA liver-directed Gene Therapy (LOPD, Phase 1) Bayer/Actus/AskBIO (Actus-101) (NCT03533673)	Myozyme/Lumizyme (IOPD, LOPD) Genzyme (alglucosidase alfa)
AAV/Proprietary capsid Gene Therapy (Amicus)	AAV9 muscle-directed Gene Therapy w/immune modulation (LOPD, Phase 1) University of Florida (NCT02240407)	Nexviazyme (LOPD) Genzyme (avalglucosidase alfa)
AAV Gene Therapy (Sarepta-licensed from Lacerta)	AAV1/CMV-hGAA muscle-directed Gene Therapy (LOPD, Phase 1/2) University of Florida (NCT00976352)	
AAV/Proprietary capsid Gene Therapy (Abeona)	AAV8 liver-directed Gene Therapy (LOPD, Phase 1/2) Audentes (AT845) (NCT04174105)	
HSPC LV Gene Therapy (Erasmus MC)	AAV/Proprietary Rh74-derived capsid, liver-directed Gene Therapy (LOPD, Phase 1/2) Spark/Roche (SPK-3006) (NCT04093349)	
HSPC LV Gene Therapy AVR-RD-03 (AVROBIO)	Chaperone/ERT (IOPD/LOPD, Phase 3) Amicus (ATB200/AT2221) (NCT03729362)	
JR-162; IV (JCR Pharma) J-Brain Cargo platform to cross blood–brain barrier	Nexviazyme (IOPD, Phase 2) Genzyme (avalglucosidase alfa) (NCT03019406)	

**Table 2:** Approved doses for LSDs. Information retrieved from accessdata.fda.gov. Burrow and Grabowski reported the U/kg to mg/kg * [10], MPS = mucopolysaccharidosis.

Disease	Product	Recommended Dosage (mg/kg)	Frequency
Fabry	FABRAZYME^®^ (agalsidase beta)	1	Every two weeks
Gaucher	VPRIV^®^ (velaglucerase alfa)	60 U/kg (~1.5 mg/kg) *	Every two weeks
MPS I	ALDURAZYME^®^ (laronidase)	0.58	Once a week
MPS II	ELAPRASE^®^ (idursulfase)	0.5	Once a week
MPS VI	NAGLAZYME^®^ (galsulfase)	1	Once a week
Pompe	MYOZYME^®^ (alglucosidase alfa)	20	Every two weeks

## Data Availability

Data is presented in Figure 4.
